# 2,4-Bis(diphenyl­phosphan­yl)-1,1,2,3,3,4-hexa­phenyl-1,3-diphospha-2,4-dibora­cyclo­butane tetra­hydro­furan sesqui­solvate

**DOI:** 10.1107/S1600536812011361

**Published:** 2012-03-21

**Authors:** Normen Peulecke, Bernd H. Müller, Anke Spannenberg, Uwe Rosenthal

**Affiliations:** a Leibniz-Institut für Katalyse e.V. an der Universität Rostock, Albert-Einstein-Strasse 29a, 18059 Rostock, Germany

## Abstract

In the title compound, C_60_H_50_B_2_P_4_·1.5C_4_H_8_O, the diphospha­diborane mol­ecule lies on an inversion centre, whereas the disordered tetra­hydro­furan solvent mol­ecule is in a general position with a partial occupancy of 0.75. The diphosphadiborane mol­ecule consists of an ideal planar four-membered B_2_P_2_ ring with an additional phenyl and a –PPh_2_ group attached to each B atom.

## Related literature

For the structure of a monomeric diphosphaborane mol­ecule, see: Bartlett *et al.* (1988 ▶). For assumed monomeric PhB(PPh_2_)_2_, see: Coates & Livingstone (1961 ▶). For the structures of other dimeric boron-bridged bis­phosphine compounds, see: Herdtweck *et al.* (1997 ▶); Kaufmann *et al.* (1997 ▶); Nöth (1987 ▶).
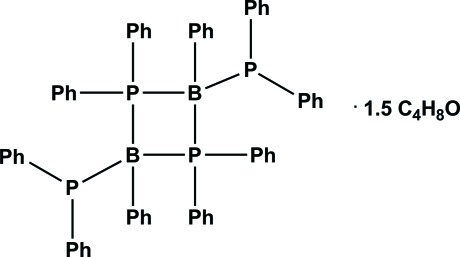


## Experimental

### 

#### Crystal data

C_60_H_50_B_2_P_4_·1.5C_4_H_8_O
*M* *_r_* = 1024.66
Orthorhombic, 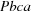

*a* = 19.2421 (4) Å
*b* = 11.6938 (2) Å
*c* = 24.9769 (5) Å
*V* = 5620.13 (19) Å^3^
*Z* = 4
Mo *K*α radiation
μ = 0.18 mm^−1^
*T* = 150 K
0.35 × 0.28 × 0.20 mm

#### Data collection

Stoe IPDS II diffractometer
Absorption correction: numerical (*X-SHAPE* and *X-RED32*; Stoe & Cie, 2005 ▶) *T*_min_ = 0.927, *T*_max_ = 0.986
91233 measured reflections
6705 independent reflections
4392 reflections with *I* > 2σ(*I*)
*R*_int_ = 0.072

#### Refinement

*R*[*F* ^2^ > 2σ(*F* ^2^)] = 0.041
*wR*(*F* ^2^) = 0.096
*S* = 0.87
6705 reflections
343 parameters
9 restraints
H-atom parameters constrained
Δρ_max_ = 0.64 e Å^−3^
Δρ_min_ = −0.29 e Å^−3^

### 

Data collection: *X-AREA* (Stoe & Cie, 2005 ▶); cell refinement: *X-AREA*; data reduction: *X-RED32* (Stoe & Cie, 2005 ▶); program(s) used to solve structure: *SHELXS97* (Sheldrick, 2008 ▶); program(s) used to refine structure: *SHELXL97* (Sheldrick, 2008 ▶); molecular graphics: *XP* in *SHELXTL* (Sheldrick, 2008 ▶); software used to prepare material for publication: *SHELXTL*.

## Supplementary Material

Crystal structure: contains datablock(s) I, global. DOI: 10.1107/S1600536812011361/yk2048sup1.cif

Structure factors: contains datablock(s) I. DOI: 10.1107/S1600536812011361/yk2048Isup2.hkl

Additional supplementary materials:  crystallographic information; 3D view; checkCIF report

## Figures and Tables

**Fig. 1.**
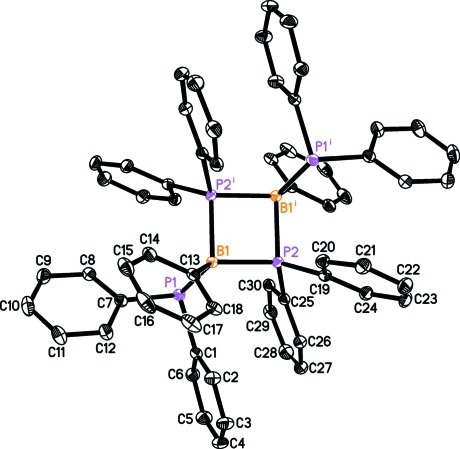
The structure of the diphosphadiborane molecule showing the atom-labelling scheme. Hydrogen atoms are omitted for clarity. Displacement ellipsoids are drawn at the 30% probability level.

## supplementary crystallographic information

### Comment

We became interested in such a class of compounds, because the boron-bridged bisphosphines could be potential ligands for the chromium catalyzed selective oligomerization of ethene, like PNP. Unfortunately the synthesis of PhB(PPh_2_)_2_, according to the literature (Coates & Livingstone, 1961), failed and always ended up with insoluble polymer. Therefore we changed the procedure using LiPPh_2_ instead of HPPh_2_. Examples of structurally characterized borone-bridged bisphosphines are known (Herdtweck *et al.*, 1997; Kaufmann *et al.*, 1997; Nöth, 1987). Only bulky substituents at the boron lead to a monomeric structure (Bartlett *et al.*, 1988). In the present publication, we report on the formation of the dimeric C_60_H_50_B_2_P_4_. In the structure of the title compound, the diphosphadiborane molecule occupies the position at an inversion center, whereas the solvent molecule of tetrahydrofuran lies in general position with partial occupancy equal to 0.75. In the tetrahydrofuran molecule the O atom is disordered over two sites with occupancies of 0.341 (9): 0.409 (9). All P—B distances in the four-membered ring are essentially identical [B1—P2 = 2.030 (3) Å and B1—P2^i^ = 2.036 (2) Å], and also the B1—P1 bond distance of 2.043 (2) Å is not significantly different. In the B_2_P_2_ ring, angles of nearly 90° were observed [P2—B1—P2^i^ = 87.24 (9)° and B1—P2—B1^i^ = 92.75°].

### Experimental

PhBCl_2_ (0.817 ml, 6.3 mmol) was added to a solution of 25 mL Ph_2_PLi (0.5*M* in thf) in 20 ml of thf at -40°C and the resulting solution was stirred at room temperature for 48 h. Subsequently, the formed light brown solution was filtered, reduced to the half, over-layered with *n*-hexane and stored at 0°C. Crystals of the title compound appeared, which were suitable for crystal structure analysis. The white compound was fully characterized by standard analytical methods *e.g.*^31^P-NMR: (C_6_D_6_): -10.7(br), -42.2(tr) p.p.m..

### Refinement

H atoms were placed in idealized positions with d(C—H) = 0.95 Å (CH), 0.99 Å (CH_2_) and refined using a riding model with *U*_iso_(H) fixed at 1.2 *U*_eq_(C).

### Crystal data

**Table:**

C_60_H_50_B_2_P_4_·1.5C_4_H_8_O	*D*_x_ = 1.211 Mg m^−^^3^
*M**_r_* = 1024.66	Mo *K*α radiation, λ = 0.71073 Å
Orthorhombic, *P**b**c**a*	Cell parameters from 6569 reflections
*a* = 19.2421 (4) Å	θ = 1.6–28.0°
*b* = 11.6938 (2) Å	µ = 0.18 mm^−^^1^
*c* = 24.9769 (5) Å	*T* = 150 K
*V* = 5620.13 (19) Å^3^	Prism, colourless
*Z* = 4	0.35 × 0.28 × 0.20 mm
*F*(000) = 2160	

### Data collection

**Table:**

Stoe IPDS II diffractometer	6705 independent reflections
Radiation source: fine-focus sealed tube	4392 reflections with *I* > 2σ(*I*)
Graphite monochromator	*R*_int_ = 0.072
ω scans	θ_max_ = 28.0°, θ_min_ = 2.2°
Absorption correction: numerical (*X-SHAPE* and *X-RED32*; Stoe & Cie, 2005)	*h* = −25→25
*T*_min_ = 0.927, *T*_max_ = 0.986	*k* = −15→15
91233 measured reflections	*l* = −32→32

### Refinement

**Table:**

Refinement on *F*^2^	Primary atom site location: structure-invariant direct methods
Least-squares matrix: full	Secondary atom site location: difference Fourier map
*R*[*F*^2^ > 2σ(*F*^2^)] = 0.041	Hydrogen site location: inferred from neighbouring sites
*wR*(*F*^2^) = 0.096	H-atom parameters constrained
*S* = 0.87	*w* = 1/[σ^2^(*F*_o_^2^) + (0.0536*P*)^2^ + 0.0*P*] where *P* = (*F*_o_^2^ + 2*F*_c_^2^)/3
6705 reflections	(Δ/σ)_max_ = 0.002
343 parameters	Δρ_max_ = 0.64 e Å^−^^3^
9 restraints	Δρ_min_ = −0.29 e Å^−^^3^

### Special details

**Table:**

Geometry. All s.u.'s (except the s.u. in the dihedral angle between two l.s. planes) are estimated using the full covariance matrix. The cell s.u.'s are taken into account individually in the estimation of s.u.'s in distances, angles and torsion angles; correlations between s.u.'s in cell parameters are only used when they are defined by crystal symmetry. An approximate (isotropic) treatment of cell s.u.'s is used for estimating s.u.'s involving l.s. planes.
Refinement. Refinement of *F*^2^ against ALL reflections. The weighted *R*-factor *wR* and goodness of fit *S* are based on *F*^2^, conventional *R*-factors *R* are based on *F*, with *F* set to zero for negative *F*^2^. The threshold expression of *F*^2^ > σ(*F*^2^) is used only for calculating *R*-factors(gt) *etc*. and is not relevant to the choice of reflections for refinement. *R*-factors based on *F*^2^ are statistically about twice as large as those based on *F*, and *R*- factors based on ALL data will be even larger.

### Fractional atomic coordinates and isotropic or equivalent isotropic displacement parameters (Å^2^)

**Table:**

	*x*	*y*	*z*	*U*_iso_*/*U*_eq_	Occ. (<1)
O1A	0.1872 (2)	0.6123 (6)	0.2276 (2)	0.061 (2)*	0.341 (9)
O1B	0.1940 (2)	0.5614 (5)	0.24577 (17)	0.0613 (19)*	0.409 (9)
C31	0.2198 (2)	0.5279 (4)	0.1944 (2)	0.0981 (17)	0.75
H31A	0.1909	0.5086	0.1629	0.118*	0.341 (9)
H31B	0.2305	0.4572	0.2147	0.118*	0.341 (9)
H31C	0.1834	0.5419	0.1672	0.118*	0.409 (9)
H31D	0.2297	0.4448	0.1948	0.118*	0.409 (9)
C32	0.2832 (2)	0.5897 (4)	0.17909 (16)	0.0913 (16)	0.75
H32A	0.2749	0.6378	0.1471	0.110*	0.75
H32B	0.3214	0.5356	0.1711	0.110*	0.75
C33	0.3004 (2)	0.6610 (4)	0.22580 (13)	0.0766 (13)	0.75
H33A	0.3059	0.7420	0.2151	0.092*	0.75
H33B	0.3441	0.6346	0.2427	0.092*	0.75
C34	0.24158 (17)	0.6479 (4)	0.26295 (13)	0.0627 (10)	0.75
H34A	0.2513	0.5891	0.2905	0.075*	0.341 (9)
H34B	0.2301	0.7211	0.2808	0.075*	0.341 (9)
H34C	0.2595	0.6279	0.2989	0.075*	0.409 (9)
H34D	0.2167	0.7217	0.2659	0.075*	0.409 (9)
C1	1.06155 (9)	−0.01162 (16)	0.34268 (7)	0.0246 (4)	
C2	1.00019 (10)	0.02724 (18)	0.31988 (7)	0.0307 (4)	
H2	0.9719	0.0795	0.3392	0.037*	
C3	0.97947 (11)	−0.00883 (19)	0.26950 (8)	0.0364 (5)	
H3	0.9371	0.0185	0.2547	0.044*	
C4	1.01996 (11)	−0.0841 (2)	0.24080 (8)	0.0369 (5)	
H4	1.0057	−0.1089	0.2063	0.044*	
C5	1.08146 (11)	−0.12344 (19)	0.26268 (8)	0.0369 (5)	
H5	1.1099	−0.1749	0.2430	0.044*	
C6	1.10164 (10)	−0.08786 (17)	0.31321 (7)	0.0308 (4)	
H6	1.1437	−0.1161	0.3280	0.037*	
C7	1.13848 (10)	0.16430 (16)	0.39173 (7)	0.0257 (4)	
C8	1.20183 (10)	0.18941 (18)	0.41622 (7)	0.0299 (4)	
H8	1.2214	0.1365	0.4408	0.036*	
C9	1.23674 (11)	0.29054 (19)	0.40521 (9)	0.0376 (5)	
H9	1.2794	0.3070	0.4228	0.045*	
C10	1.20972 (12)	0.36709 (19)	0.36884 (9)	0.0414 (5)	
H10	1.2332	0.4369	0.3617	0.050*	
C11	1.14820 (11)	0.34169 (18)	0.34279 (9)	0.0383 (5)	
H11	1.1302	0.3933	0.3169	0.046*	
C12	1.11277 (10)	0.24195 (18)	0.35416 (8)	0.0319 (4)	
H12	1.0704	0.2259	0.3362	0.038*	
C13	0.98211 (9)	0.19751 (16)	0.43708 (7)	0.0240 (4)	
C14	1.01833 (11)	0.29722 (16)	0.45140 (7)	0.0304 (4)	
H14	1.0579	0.2909	0.4740	0.036*	
C15	0.99832 (12)	0.40388 (18)	0.43376 (9)	0.0390 (5)	
H15	1.0236	0.4696	0.4448	0.047*	
C16	0.94210 (12)	0.41587 (19)	0.40029 (9)	0.0427 (6)	
H16	0.9291	0.4892	0.3874	0.051*	
C17	0.90491 (12)	0.32050 (19)	0.38570 (8)	0.0382 (5)	
H17	0.8659	0.3282	0.3627	0.046*	
C18	0.92386 (10)	0.21293 (17)	0.40430 (7)	0.0280 (4)	
H18	0.8967	0.1484	0.3946	0.034*	
C19	0.86269 (9)	−0.06475 (16)	0.46070 (6)	0.0237 (4)	
C20	0.82663 (9)	0.03413 (16)	0.47541 (7)	0.0263 (4)	
H20	0.8517	0.1014	0.4844	0.032*	
C21	0.75461 (10)	0.03493 (18)	0.47697 (8)	0.0314 (4)	
H21	0.7306	0.1022	0.4876	0.038*	
C22	0.71758 (10)	−0.06199 (19)	0.46314 (8)	0.0344 (4)	
H22	0.6682	−0.0612	0.4639	0.041*	
C23	0.75253 (10)	−0.15974 (18)	0.44826 (8)	0.0328 (4)	
H23	0.7271	−0.2259	0.4381	0.039*	
C24	0.82462 (10)	−0.16246 (17)	0.44795 (7)	0.0280 (4)	
H24	0.8482	−0.2313	0.4390	0.034*	
C25	0.98791 (10)	−0.18291 (16)	0.42454 (7)	0.0243 (4)	
C26	0.95519 (11)	−0.21012 (17)	0.37607 (7)	0.0295 (4)	
H26	0.9138	−0.1714	0.3660	0.035*	
C27	0.98292 (11)	−0.29323 (18)	0.34282 (8)	0.0359 (5)	
H27	0.9600	−0.3124	0.3104	0.043*	
C28	1.04372 (12)	−0.34830 (17)	0.35668 (8)	0.0385 (5)	
H28	1.0624	−0.4054	0.3338	0.046*	
C29	1.07740 (11)	−0.32094 (17)	0.40355 (8)	0.0337 (5)	
H29	1.1197	−0.3581	0.4126	0.040*	
C30	1.04967 (10)	−0.23923 (16)	0.43754 (7)	0.0268 (4)	
H30	1.0729	−0.2213	0.4700	0.032*	
B1	1.01311 (10)	0.07717 (18)	0.45461 (7)	0.0212 (4)	
P1	1.09614 (2)	0.02764 (4)	0.409099 (17)	0.02250 (11)	
P2	0.95699 (2)	−0.06707 (4)	0.467327 (17)	0.02053 (10)	

### Atomic displacement parameters (Å^2^)

**Table:**

	*U*^11^	*U*^22^	*U*^33^	*U*^12^	*U*^13^	*U*^23^
C31	0.079 (3)	0.089 (4)	0.126 (4)	−0.017 (3)	−0.022 (3)	−0.053 (3)
C32	0.098 (4)	0.128 (4)	0.048 (2)	−0.003 (3)	0.015 (2)	−0.035 (3)
C33	0.081 (3)	0.080 (3)	0.069 (3)	−0.027 (2)	0.015 (2)	−0.019 (2)
C34	0.048 (2)	0.089 (3)	0.0510 (19)	−0.013 (2)	0.0032 (16)	−0.0253 (19)
C1	0.0280 (9)	0.0259 (10)	0.0200 (8)	−0.0036 (8)	0.0021 (7)	0.0005 (7)
C2	0.0311 (10)	0.0355 (11)	0.0256 (9)	0.0051 (9)	−0.0015 (8)	−0.0013 (8)
C3	0.0369 (11)	0.0464 (13)	0.0260 (9)	0.0042 (10)	−0.0062 (8)	0.0006 (9)
C4	0.0420 (12)	0.0480 (13)	0.0207 (9)	−0.0042 (10)	−0.0003 (8)	−0.0058 (9)
C5	0.0399 (12)	0.0422 (13)	0.0285 (10)	0.0029 (10)	0.0061 (8)	−0.0082 (9)
C6	0.0287 (10)	0.0343 (11)	0.0294 (9)	0.0007 (9)	0.0010 (8)	−0.0022 (8)
C7	0.0261 (9)	0.0268 (10)	0.0243 (9)	−0.0006 (8)	0.0043 (7)	−0.0015 (7)
C8	0.0293 (10)	0.0337 (11)	0.0267 (9)	−0.0032 (8)	0.0019 (8)	0.0003 (8)
C9	0.0321 (11)	0.0420 (12)	0.0387 (11)	−0.0092 (9)	0.0044 (9)	−0.0064 (10)
C10	0.0395 (12)	0.0311 (12)	0.0535 (13)	−0.0086 (10)	0.0155 (10)	0.0006 (10)
C11	0.0370 (12)	0.0322 (12)	0.0458 (12)	0.0028 (9)	0.0100 (10)	0.0113 (9)
C12	0.0271 (10)	0.0330 (11)	0.0355 (10)	−0.0004 (8)	0.0028 (8)	0.0068 (9)
C13	0.0284 (9)	0.0237 (9)	0.0199 (8)	0.0006 (8)	0.0053 (7)	0.0009 (7)
C14	0.0389 (11)	0.0246 (10)	0.0276 (9)	−0.0031 (9)	0.0080 (8)	0.0011 (8)
C15	0.0532 (13)	0.0237 (11)	0.0401 (11)	−0.0005 (10)	0.0192 (10)	0.0018 (9)
C16	0.0525 (13)	0.0302 (12)	0.0455 (12)	0.0149 (10)	0.0232 (10)	0.0159 (10)
C17	0.0359 (11)	0.0462 (13)	0.0324 (10)	0.0167 (10)	0.0111 (9)	0.0150 (9)
C18	0.0290 (10)	0.0321 (10)	0.0229 (8)	0.0056 (8)	0.0054 (7)	0.0043 (8)
C19	0.0248 (8)	0.0265 (9)	0.0198 (8)	−0.0023 (8)	−0.0019 (7)	0.0022 (7)
C20	0.0278 (9)	0.0252 (10)	0.0257 (8)	−0.0013 (8)	−0.0036 (7)	0.0019 (7)
C21	0.0294 (10)	0.0317 (11)	0.0331 (10)	0.0033 (8)	−0.0045 (8)	0.0013 (8)
C22	0.0230 (9)	0.0424 (12)	0.0379 (10)	−0.0015 (9)	−0.0057 (8)	0.0018 (9)
C23	0.0290 (10)	0.0330 (11)	0.0363 (10)	−0.0093 (9)	−0.0073 (8)	0.0008 (9)
C24	0.0289 (10)	0.0267 (10)	0.0285 (9)	−0.0021 (8)	−0.0035 (8)	0.0012 (8)
C25	0.0300 (10)	0.0207 (9)	0.0221 (8)	−0.0051 (8)	0.0038 (7)	−0.0002 (7)
C26	0.0335 (10)	0.0294 (10)	0.0255 (9)	−0.0058 (9)	0.0008 (8)	−0.0008 (7)
C27	0.0471 (13)	0.0333 (11)	0.0271 (9)	−0.0123 (10)	0.0023 (9)	−0.0075 (8)
C28	0.0524 (13)	0.0265 (11)	0.0367 (11)	−0.0033 (10)	0.0130 (10)	−0.0102 (9)
C29	0.0394 (11)	0.0244 (10)	0.0375 (11)	0.0005 (8)	0.0080 (9)	0.0003 (8)
C30	0.0306 (10)	0.0236 (10)	0.0263 (9)	−0.0034 (8)	0.0037 (8)	0.0015 (7)
B1	0.0235 (9)	0.0220 (10)	0.0182 (9)	−0.0021 (8)	−0.0006 (7)	0.0005 (7)
P1	0.0239 (2)	0.0231 (2)	0.0206 (2)	0.0002 (2)	−0.00034 (18)	0.00079 (18)
P2	0.0229 (2)	0.0195 (2)	0.01925 (19)	−0.00212 (19)	−0.00134 (18)	−0.00060 (17)

### Geometric parameters (Å, º)

**Table:**

O1A—C34	1.431 (3)	C12—H12	0.9500
O1A—C31	1.433 (4)	C13—C18	1.400 (3)
O1B—C34	1.429 (3)	C13—C14	1.405 (3)
O1B—C31	1.430 (4)	C13—B1	1.590 (3)
C31—C32	1.470 (3)	C14—C15	1.378 (3)
C31—H31A	0.9900	C14—H14	0.9500
C31—H31B	0.9900	C15—C16	1.374 (3)
C31—H31C	0.9900	C15—H15	0.9500
C31—H31D	0.9900	C16—C17	1.374 (3)
C32—C33	1.471 (3)	C16—H16	0.9500
C32—H32A	0.9900	C17—C18	1.390 (3)
C32—H32B	0.9900	C17—H17	0.9500
C33—C34	1.472 (3)	C18—H18	0.9500
C33—H33A	0.9900	C19—C24	1.394 (3)
C33—H33B	0.9900	C19—C20	1.398 (3)
C34—H34A	0.9900	C19—P2	1.8222 (18)
C34—H34B	0.9900	C20—C21	1.386 (3)
C34—H34C	0.9900	C20—H20	0.9500
C34—H34D	0.9900	C21—C22	1.383 (3)
C1—C2	1.387 (3)	C21—H21	0.9500
C1—C6	1.390 (3)	C22—C23	1.377 (3)
C1—P1	1.8455 (18)	C22—H22	0.9500
C2—C3	1.386 (3)	C23—C24	1.388 (3)
C2—H2	0.9500	C23—H23	0.9500
C3—C4	1.377 (3)	C24—H24	0.9500
C3—H3	0.9500	C25—C30	1.397 (3)
C4—C5	1.382 (3)	C25—C26	1.401 (2)
C4—H4	0.9500	C25—P2	1.8251 (19)
C5—C6	1.385 (3)	C26—C27	1.385 (3)
C5—H5	0.9500	C26—H26	0.9500
C6—H6	0.9500	C27—C28	1.380 (3)
C7—C8	1.395 (3)	C27—H27	0.9500
C7—C12	1.396 (3)	C28—C29	1.376 (3)
C7—P1	1.8454 (19)	C28—H28	0.9500
C8—C9	1.388 (3)	C29—C30	1.385 (3)
C8—H8	0.9500	C29—H29	0.9500
C9—C10	1.377 (3)	C30—H30	0.9500
C9—H9	0.9500	B1—P2	2.028 (2)
C10—C11	1.383 (3)	B1—P2^i^	2.0361 (18)
C10—H10	0.9500	B1—P1	2.045 (2)
C11—C12	1.381 (3)	P2—B1^i^	2.0363 (18)
C11—H11	0.9500		
			
C34—O1A—C31	103.7 (3)	C9—C10—H10	120.2
C34—O1B—C31	104.0 (3)	C11—C10—H10	120.2
O1B—C31—C32	112.7 (3)	C12—C11—C10	120.5 (2)
O1A—C31—C32	100.1 (4)	C12—C11—H11	119.8
O1B—C31—H31A	125.6	C10—C11—H11	119.8
O1A—C31—H31A	111.8	C11—C12—C7	120.84 (19)
C32—C31—H31A	111.8	C11—C12—H12	119.6
O1B—C31—H31B	81.0	C7—C12—H12	119.6
O1A—C31—H31B	111.8	C18—C13—C14	116.06 (17)
C32—C31—H31B	111.8	C18—C13—B1	125.14 (17)
H31A—C31—H31B	109.5	C14—C13—B1	118.58 (16)
O1B—C31—H31C	109.0	C15—C14—C13	122.1 (2)
O1A—C31—H31C	88.5	C15—C14—H14	119.0
C32—C31—H31C	109.0	C13—C14—H14	119.0
H31B—C31—H31C	129.5	C16—C15—C14	120.5 (2)
O1B—C31—H31D	109.0	C16—C15—H15	119.8
O1A—C31—H31D	138.9	C14—C15—H15	119.8
C32—C31—H31D	109.0	C15—C16—C17	119.23 (19)
H31A—C31—H31D	83.9	C15—C16—H16	120.4
H31C—C31—H31D	107.8	C17—C16—H16	120.4
C31—C32—C33	105.0 (3)	C16—C17—C18	120.6 (2)
C31—C32—H32A	110.8	C16—C17—H17	119.7
C33—C32—H32A	110.8	C18—C17—H17	119.7
C31—C32—H32B	110.8	C17—C18—C13	121.5 (2)
C33—C32—H32B	110.8	C17—C18—H18	119.3
H32A—C32—H32B	108.8	C13—C18—H18	119.3
C32—C33—C34	105.6 (3)	C24—C19—C20	118.51 (16)
C32—C33—H33A	110.6	C24—C19—P2	122.12 (14)
C34—C33—H33A	110.6	C20—C19—P2	118.87 (14)
C32—C33—H33B	110.6	C21—C20—C19	120.61 (18)
C34—C33—H33B	110.6	C21—C20—H20	119.7
H33A—C33—H33B	108.8	C19—C20—H20	119.7
O1B—C34—C33	112.1 (3)	C22—C21—C20	120.17 (19)
O1A—C34—C33	101.7 (3)	C22—C21—H21	119.9
O1B—C34—H34A	80.7	C20—C21—H21	119.9
O1A—C34—H34A	111.4	C23—C22—C21	119.75 (18)
C33—C34—H34A	111.4	C23—C22—H22	120.1
O1B—C34—H34B	127.1	C21—C22—H22	120.1
O1A—C34—H34B	111.4	C22—C23—C24	120.57 (18)
C33—C34—H34B	111.4	C22—C23—H23	119.7
H34A—C34—H34B	109.3	C24—C23—H23	119.7
O1B—C34—H34C	109.2	C23—C24—C19	120.35 (18)
O1A—C34—H34C	138.2	C23—C24—H24	119.8
C33—C34—H34C	109.2	C19—C24—H24	119.8
H34B—C34—H34C	82.8	C30—C25—C26	118.43 (17)
O1B—C34—H34D	109.2	C30—C25—P2	119.41 (13)
O1A—C34—H34D	86.8	C26—C25—P2	121.88 (15)
C33—C34—H34D	109.2	C27—C26—C25	120.28 (19)
H34A—C34—H34D	130.1	C27—C26—H26	119.9
H34C—C34—H34D	107.9	C25—C26—H26	119.9
C2—C1—C6	117.71 (17)	C28—C27—C26	120.24 (19)
C2—C1—P1	126.48 (14)	C28—C27—H27	119.9
C6—C1—P1	115.81 (14)	C26—C27—H27	119.9
C3—C2—C1	121.20 (19)	C29—C28—C27	120.29 (19)
C3—C2—H2	119.4	C29—C28—H28	119.9
C1—C2—H2	119.4	C27—C28—H28	119.9
C4—C3—C2	120.30 (19)	C28—C29—C30	120.0 (2)
C4—C3—H3	119.9	C28—C29—H29	120.0
C2—C3—H3	119.9	C30—C29—H29	120.0
C3—C4—C5	119.42 (18)	C29—C30—C25	120.69 (18)
C3—C4—H4	120.3	C29—C30—H30	119.7
C5—C4—H4	120.3	C25—C30—H30	119.7
C4—C5—C6	120.05 (19)	C13—B1—P2	125.41 (13)
C4—C5—H5	120.0	C13—B1—P2^i^	114.90 (12)
C6—C5—H5	120.0	P2—B1—P2^i^	87.27 (8)
C5—C6—C1	121.32 (19)	C13—B1—P1	113.00 (12)
C5—C6—H6	119.3	P2—B1—P1	105.52 (9)
C1—C6—H6	119.3	P2^i^—B1—P1	107.17 (9)
C8—C7—C12	117.86 (18)	C7—P1—C1	99.40 (8)
C8—C7—P1	117.71 (14)	C7—P1—B1	103.32 (8)
C12—C7—P1	124.38 (15)	C1—P1—B1	106.76 (8)
C9—C8—C7	121.03 (19)	C19—P2—C25	106.41 (9)
C9—C8—H8	119.5	C19—P2—B1	120.25 (8)
C7—C8—H8	119.5	C25—P2—B1	110.61 (8)
C10—C9—C8	120.13 (19)	C19—P2—B1^i^	111.67 (8)
C10—C9—H9	119.9	C25—P2—B1^i^	115.19 (8)
C8—C9—H9	119.9	B1—P2—B1^i^	92.73 (8)
C9—C10—C11	119.6 (2)		

Symmetry code: (i) −*x*+2, −*y*, −*z*+1.
